# A Novel Probability Model for LncRNA–Disease Association Prediction Based on the Naïve Bayesian Classifier

**DOI:** 10.3390/genes9070345

**Published:** 2018-07-08

**Authors:** Jingwen Yu, Pengyao Ping, Lei Wang, Linai Kuang, Xueyong Li, Zhelun Wu

**Affiliations:** 1 Key Laboratory of Intelligent Computing & Information Processing, Xiangtan University, Xiangtan 411105, China; jingwen.yu18@gmail.com (J.Y.); pengyao.ping2017@gmail.com (P.P.); kla@xtu.edu.cn (L.K.); 2 College of Computer Engineering & Applied Mathematics, Changsha University, Changsha 410001, China; xueyong.li111@gmail.com; 3 Department of Computer Science, Princeton University, Princeton, NJ 08544, USA; zhelunw@princeton.edu

**Keywords:** lncRNA–disease associations, tripartite network, quadruple network, prediction model, Naïve Bayesian Classifier

## Abstract

An increasing number of studies have indicated that long-non-coding RNAs (lncRNAs) play crucial roles in biological processes, complex disease diagnoses, prognoses, and treatments. However, experimentally validated associations between lncRNAs and diseases are still very limited. Recently, computational models have been developed to discover potential associations between lncRNAs and diseases by integrating multiple heterogeneous biological data; this has become a hot topic in biological research. In this article, we constructed a global tripartite network by integrating a variety of biological information including miRNA–disease, miRNA–lncRNA, and lncRNA–disease associations and interactions. Then, we constructed a global quadruple network by appending gene–lncRNA interaction, gene–disease association, and gene–miRNA interaction networks to the global tripartite network. Subsequently, based on these two global networks, a novel approach was proposed based on the naïve Bayesian classifier to predict potential lncRNA–disease associations (NBCLDA). Comparing with the state-of-the-art methods, our new method does not entirely rely on known lncRNA–disease associations, and can achieve a reliable performance with effective area under ROC curve (AUCs)in leave-one-out cross validation. Moreover, in order to further estimate the performance of NBCLDA, case studies of colorectal cancer, prostate cancer, and glioma were implemented in this paper, and the simulation results demonstrated that NBCLDA can be an excellent tool for biomedical research in the future.

## 1. Introduction

Long non-coding RNAs (lncRNAs), those with over 200 nucleotides in length [1,2,3], are considered a new class of non-protein-coding transcripts. Much research evidence has shown that lncRNAs participate in almost the entire cell life cycle through various mechanisms and play significant roles in multiple biological processes including transcription, translation, epigenetic regulation, splicing, differentiation, immune response, cell cycle control, and so on [4,5,6,7,8]. In particular, the mutations and dysregulations of lncRNAs have been proven to be closely related to various human complex diseases [9,10,11], including AIDS [12], diabetes [13], Alzheimer’s Disease (AD) [14], and many types of cancers such as breast [15], prostate [16], hepatocellular [17], and bladder cancer [18]. For instance, the expression of the lncRNA called HOTAIR was shown to be higher in primary breast tumors and metastases, and the HOTAIR expression level was proven to be a powerful predictor of eventual metastasis and death [19,20]. Additionally, the lncRNA MALAT1 was demonstrated as a prognostic indicator as well as a therapeutic target and acts as a potential therapeutic method for preventing lung cancer metastasis, which is targeted by antisense oligonucleotides (ASO) [21]. Moreover, recent studies have shown that the human H19 gene is frequently overexpressed in the myometrium and stroma during pathological endometrial proliferative events [22].

Obviously, predicting potential associations between lncRNAs and diseases would contribute to systematically understanding the pathogenesis of complex diseases at the molecular level and facilitate the identification of biomarkers for disease diagnosis, treatment, and prediction of response to therapy. However, relatively few experiments have supported lncRNA–disease associations until now. Hence, developing effective computational methods to uncover the potential associations between lncRNAs and diseases has become a hot topic in recent years. In general, existing models for predicting potential associations between lncRNAs and diseases can be divided into three categories. Among them, the first kind of methods are based on known disease-related lncRNAs. For example, Sun et al. proposed a model named RWRlncD [23], which carried out a random walk with the restart method on an lncRNA functional similarity network. This method uncovered potential associations between lncRNAs and diseases by integrating the disease similarity network, the lncRNAs functional network, and known lncRNA–disease associations. Ping et al. developed a method based on a newly constructed bipartite network, which relies on the known associations between lncRNAs and diseases [24]. Yang et al. constructed a coding-non-coding gene–disease bipartite network based on known associations between diseases and disease-causing genes (including lncRNAs). Then, they developed an iterative algorithm to uncover the possible links in the newly constructed bipartite network [25]. Ding et al. proposed a new model named TPGLDA to predict potential lncRNA–disease associations by integrating gene–disease associations with lncRNA–disease associations [26].

Different from the first kind of methods based on known lncRNA–disease associations, the second category of prediction models does not rely on known disease-related lncRNAs. For example, Chen et al. proposed a new method called HGLDA by integrating micro-RNA (miRNA)–disease associations and lncRNA–miRNA interactions. A hypergeometric distribution test is then applied to identify potential lncRNA–disease associations [27]. Liu et al. developed a computational framework by integrating human lncRNA expression profiles, gene expression profiles, and human disease-associated gene data to predict potential human lncRNA–disease associations [28]. Li et al. put forward a prediction method on account of the information of genome location to globally discover potential human lncRNAs related to vascular disease [29]. Gu et al. proposed a random walk-based model to identify potential associations between lncRNAs and diseases, which can be applied for predicting a disease without known associated lncRNAs and for inferring an lncRNA without known associated diseases [30].

In recent years, an increasing number of studies have been developed for understanding the cellular process, molecular interactions, and the pathogenesis of complex diseases at the molecular level by integrating different types of data and molecular interaction networks [31]. Such research includes the prediction of gene–disease associations [32], and the prediction of potential disease-associated miRNAs [33,34]. An increasing number of researchers have also adopted various data frameworks to increase the reliability of association prediction between diseases and lncRNAs. Hence, a third kind of prediction models has been proposed, in which multiple data sources are integrated to identify disease-related lncRNAs. For example, Lu et al. proposed a new prediction of lncRNA–disease associations via inductive matrix completion (named SIMCLDA), by integrating known lncRNA–disease interactions, disease–gene, gene–gene ontology associations [35]. Zhang et al. developed a novel model named LncRDNetFlow, which utilized a flow propagation algorithm to integrate a variety of information including the similarity of lncRNAs, the protein–protein interactions, and the similarity of diseases to infer lncRNA–disease associations [36]. Fu et al. proposed a model called MFLDA to predict potential lncRNA–disease associations by considering the quality and relevance of different heterogeneous data sources, which can select and integrate the data sources by assigning different weights to them [37]. Chen developed a path-based approach named KATZLDA for discovering potential lncRNA–disease associations by integrating information including known lncRNA–disease associations, lncRNA expression profiles, lncRNA functional similarity, disease semantic similarity, and Gaussian interaction profile kernel similarity [38]. All of these above data fusion-based methods can achieve effective results.

In this paper, to effectively predict potential lncRNA–disease associations, we first constructed a global tripartite network by integrating three kinds of heterogeneous networks including an lncRNA–disease association network, an miRNA–disease association network, and an miRNA–lncRNA interaction network. Then, considering that more heterogeneous networks can boost the prediction performance, we constructed a quadruple global network by appending a gene–lncRNA interaction network, a gene–disease association network, and a gene–miRNA interaction network to the tripartite network. Thereafter, based on these two newly constructed global networks, a novel probabilistic model named Naïve Bayesian Classifier used to predict potential LncRNA–Disease Associations (NBCLDA), based on the naïve Bayesian classifier, is proposed to uncover potential lncRNA–disease associations. Moreover, in order to evaluate the prediction performance of the NBCLDA, the leave-one-out cross-validation (LOOCV) framework was implemented, and the experimental results demonstrated the effective performance of the NBCLDA and illustrated that it can achieve better predictive performance than state-of-the-art methods in the terms of LOOCV.

## 2. Data Collection and Preprocessing

Considering that more heterogeneous data sources can boost the performance of prediction models, in this paper, to construct our novel prediction model NBCLDA—with the ultimate goal being to infer potential associations between lncRNAs and diseases-seven heterogeneous data sets were combined. These include the sets of miRNA–disease, miRNA–lncRNA, lncRNA–disease, gene–disease, and gene–lncRNA associations, as well as the sets of gene–miRNA interactions, and of diseases with disease tree numbers. The sets were collected from various databases.

### 2.1. Construction of miRNA–Disease and miRNA–lncRNA Association Sets

In this article, the miRNA–disease and miRNA–lncRNA association sets were downloaded from the HMDD [39] and the starBase v2.0 [40] databases in January 2015. Once these two data sets were collected, we removed any duplicate associations with conflicting evidence. Then, we further unified the names of miRNAs, and, thereafter, manually selected the common miRNAs in both sets. Finally, we retained only the associations related with those selected miRNAs in these two data sets. As a result, we obtained a data set ${\mathrm{DS}}_{1}$ consisting of 4704 miRNA–disease interactions between 246 miRNAs and 373 diseases, and a data set ${\mathrm{DS}}_{2}$ consisting of 9086 miRNA–lncRNA interactions between 246 miRNAs and 1089 lncRNAs (see Supplementary Materials Tables S1 and S2).

### 2.2. Construction of the lncRNA–Disease Association Set

In this paper, the set of lncRNA–disease associations was collected from the MNDR v2.0 database [41] in 2017. In a similar way, once the data set was collected, we removed the duplicate associations with conflicting evidence. Then, we selected the lncRNA–disease associations with diseases belonging to ${\mathrm{DS}}_{1}$ and lncRNAs belonging to ${\mathrm{DS}}_{2}$ simultaneously. As a result, we obtained a data set ${\mathrm{DS}}_{3}$ consisting of 407 lncRNA–disease associations between 77 lncRNAs and 95 diseases (see Supplementary Materials Table S3). The data set ${\mathrm{DS}}_{3}$ is utilized as the test sample in our following simulation experiments.

### 2.3. Construction of the Gene–Disease and Gene–lncRNA Association Sets

In this article, the set of gene–disease associations was gathered from the DisGeNET v5.0 database [42] in May 2017, and the set of gene–lncRNA associations was downloaded from the LncACTdb v1.0 database [43]. Again, we removed the duplicate associations with conflicting evidence. Then, we further unified the names of genes, and thereafter manually selected the common genes in both sets. Finally, we retained only the associations related with those selected genes in these two data sets. Additionally, we transformed some disease names included in the newly constructed set of gene–disease associations into their aliases in the ${\mathrm{DS}}_{1}$, in order to keep the uniformity of disease names. For example, the disease names “pulmonary Emphysema” and “Bladder Neoplasm” in the newly collected set of gene–disease associations was converted into “pulmonary Embolism” and “Bladder Neoplasms” in the ${\mathrm{DS}}_{1}$, respectively. Hence, we obtained a data set ${\mathrm{DS}}_{4}$ consisting of 3702 gene–disease associations between 171 genes and 227 diseases, and a data set ${\mathrm{DS}}_{5}$ consisting of 411 gene–lncRNA interactions between 171 genes and 66 lncRNAs (see Supplementary Materials Tables S4 and S5).

### 2.4. Construction of the Gene–miRNA Association Set

In this paper, the set of gene–miRNA interactions was obtained from the miRecords [44] database that was last updated in April 2013. Once the data set was collected, we removed the duplicate associations with conflicting evidence. Then, we selected the gene–miRNA interactions with genes belonging to ${\mathrm{DS}}_{4}$ or ${\mathrm{DS}}_{5}$ and miRNAs belonging to ${\mathrm{DS}}_{1}$ or ${\mathrm{DS}}_{2}$, simultaneously. Finally, as a result, we obtained a data set ${\mathrm{DS}}_{6}$ consisting of 565 gene–miRNA associations between 109 genes and 174 miRNAs (see Supplementary Materials Table S6).

### 2.5. Construction of the Set of Diseases with Disease Tree Numbers

In this article, the set of diseases with Disease tree numbers was gathered from the MeSH database [45] . In the MeSH database, the disease terms, described as DAGs, were classified and signified as disease tree numbers. We browsed the MeSH database and collected the disease tree numbers of diseases in ${\mathrm{DS}}_{1}$. As a result, we obtained a data set ${\mathrm{DS}}_{7}$ consisting of 373 diseases with their disease tree numbers (see Supplementary Materials Table S7).

### 2.6. Analysis of Multi Relational Data Sources

**Figure 1:**
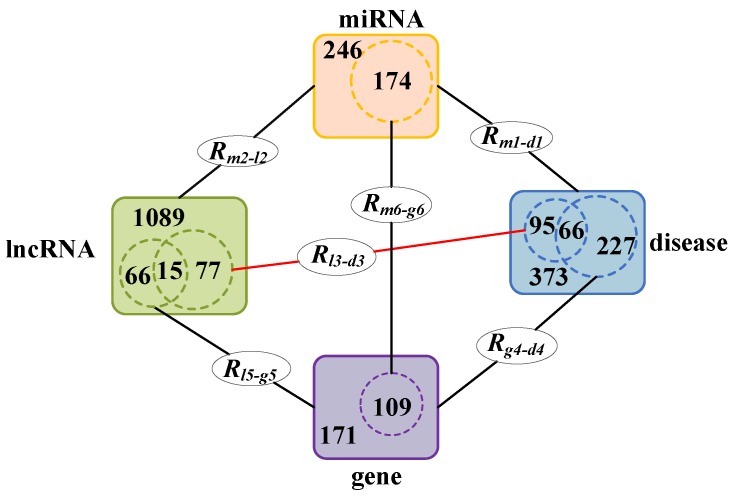
The relationship between the different data sources and number of data points.

In our model, four object types such as lncRNA, diseases, miRNA, and genes are considered. Based on these four object types, we collect six relational data sources from different databases. Figure 1 is constructed to illustrate the relationship between these different data sources more directly. In Figure 1, $R_{{\#}_{1}\Omega -{\#}_{2}\Omega }$ denotes the different associations between these four object types, where ${\#}_{1}$ represents one object, ${\#}_{2}$ represents another object and Ω denotes the dataset ${\mathrm{DS}}_{\Omega }$ that the two objects belong to. For example, $R_{m1-d1}$ denotes the associations between miRNAs and diseases, *m* represents miRNAs, d represents diseases, and ‘1’ indicates all these miRNAs and diseases belong to the dataset ${\mathrm{DS}}_{1}$. In addition, the numbers of the same objects in the different datasets and the relationships among them are shown in Figure 1. For instance, the number of diseases is 373 in $R_{m1-d1}$, 95 (= 29 + 66) in $R_{l3-d3}$ and 227 (= 66 + 161) in $R_{g4-d4}$, and it is obvious that both the 95 diseases in $R_{l3-d3}$ and the 227 diseases in $R_{g4-d4}$ are part of the 373 diseases in $R_{m1-d1}$; moreover, the intersect of disease in $R_{l3-d3}$ and $R_{g4-d4}$ includes 66 different diseases.

## 3. Method

**Figure 2:**
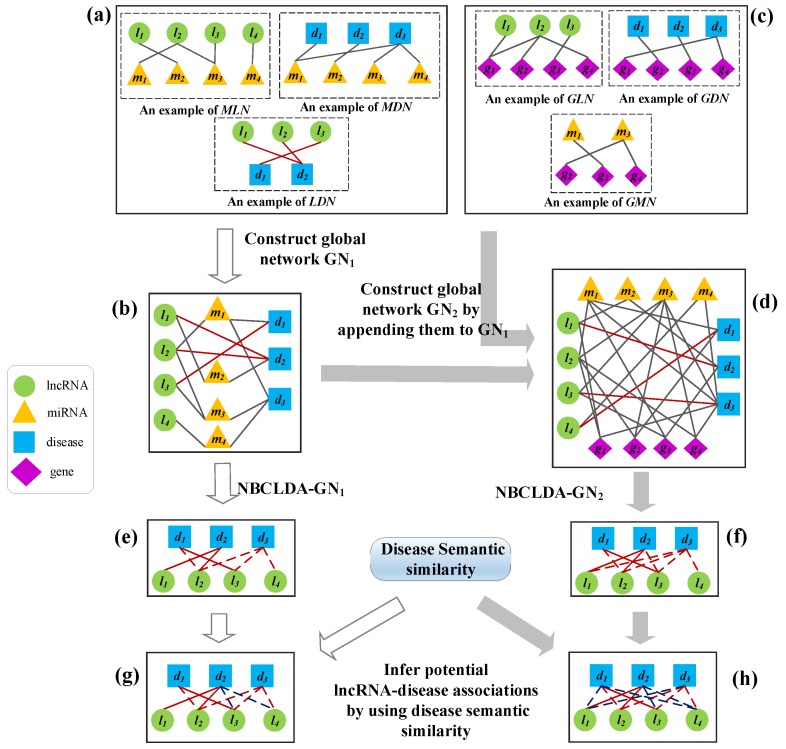
The flowchart of NBCLDA. In the diagram, the green circles, blue squares, orange triangles, and purple diamonds represent lncRNAs, diseases, miRNAs, and genes, respectively. (**a**) construction of the MDN, MLN, and LDN; (**b**) construction of global tripartite network $GN_{1}$ by integrating the MDN, MLN, and LDN; (**c**) construction of the GDN, GLN, and GMN; (**d**) construction of the global quadruple network $GN_{2}$ by appending the GDN, GLN, and GMN into $GN_{1}$; (**e**,**f**) construction of the potential lncRNA–disease association network by using the NBCLDA-$GN_{1}$, and NBCLDA-$GN_{2}$; (**g**,**h**) inference of potential lncRNA–disease associations by using disease semantic similarity. Here, in (**e**–**h**), the known lncRNA–disease associations are represented as the solid edges, and the candidate lncRNA–disease associations are represented as dashed edges.

As illustrated in Figure 2, our newly proposed model NBCLDA for predicting potential associations between lncRNAs and diseases can be mainly divided into the following steps:

Step 1: As illustrated in Figure 2a, on the basis of data sets ${\mathrm{DS}}_{1}$, ${\mathrm{DS}}_{2}$, and ${\mathrm{DS}}_{3}$ we can construct an miRNA–disease association network labeled MDN, an miRNA–lncRNA association network labeled MLN, and an lncRNA–disease association network labeled LDN.

Step 2: As illustrated in Figure 2b, by integrating the three association networks constructed in Step 1, we can easily obtain a global tripartite network $GN_{1}$ of lncRNA–miRNA–disease relationships.

Step 3: As illustrated in Figure 2c, in order to utilize multiple data sources to improve the prediction performance, on the basis of data sets ${\mathrm{DS}}_{4}$, ${\mathrm{DS}}_{5}$, and ${\mathrm{DS}}_{6}$ obtained above, we can also construct a gene–disease association network labeled GDN, a gene–lncRNA association network labeled GLN, and a gene–miRNA association network labeled GMN.

Step 4: As illustrated in Figure 2d, by appending the three association networks constructed in Step 3 to $GN_{1}$ constructed in Step 2, we can easily obtain a global quadruple network $GN_{2}$ of lncRNA–miRNA–gene–disease relations.

Step 5: As illustrated in Figure 2e,f, after applying the naïve Bayesian classifier theory to $GN_{1}$ and $GN_{2}$, we can obtain two kinds of prediction models: NBCLDA-$GN_{1}$ and NBCLDA-$GN_{2}$.

Step 6: As illustrated in Figure 2g,h, in order to further improve the prediction performance of the NBCLDA, we implemented disease semantic similarity in NBCLDA-$GN_{1}$ and NBCLDA-$GN_{2}$. Thus, we can obtain two new prediction models, NBCLDA-$GN_{1}$-SD and NBCLDA-$GN_{2}$-SD, to infer potential lncRNA–disease associations.

### 3.1. Construction of the MDN, MLN, LDN, and $GN_{1}$

Let *L* be the set of *n* lncRNAs in ${\mathrm{DS}}_{2}$, $L^{\prime }$ be the set of $n^{\prime }$ lncRNAs in ${\mathrm{DS}}_{3}$, *D* be the set of *r* diseases in ${\mathrm{DS}}_{1}$, $D^{\prime }$ be the set of $r^{\prime }$ diseases in ${\mathrm{DS}}_{3}$. Additionally, let $M=\{m_{1},m_{2},\ldots ,m_{t}\}$ be the set of *t* miRNAs in ${\mathrm{DS}}_{1}$ or ${\mathrm{DS}}_{2}$. From Section 2.1 and Section 2.2, it is clear that $L^{\prime }\subseteq L$ and $D^{\prime }\subseteq D$; hence, we can let $L^{\prime }=\left\{l_{1},l_{2},\ldots ,l_{n^{\prime }}\right\}$, $L=\{l_{1},l_{2},\ldots ,l_{n^{\prime }},l_{n^{\prime }+1},\ldots ,l_{n}\}$, $D^{\prime }=\left\{d_{1},d_{2},\ldots ,d_{r^{\prime }}\right\}$, and $D=\{d_{1},d_{2},\ldots ,d_{r^{\prime }},d_{r^{\prime }+1},\ldots ,d_{r}\}$. Thus, we can represent the miRNA–disease association network, MDN, as $MDN=(M,D,E_{1})$, where $E_{1}=\left\{e_{m_{k}-d_{j}}|m_{k}\in M,d_{j}\in D\right\}$ denotes the set of known interactions between the miRNAs in *M* and the diseases in *D*. That is, the edge $e_{m_{k}-d_{j}}\in E_{1}\Leftrightarrow m_{k}$ is associated with $d_{j}$.

In the same way, we can further represent the miRNA–lncRNA interaction network, MLN, and the lncRNA–disease association network, LDN, as $MDN=(M,L,E_{2})$ and $LDN=(L,D,E_{3})$, where $E_{2}=\left\{e_{m_{k}-l_{i}}|m_{k}\in M,l_{i}\in L\right\}$ denotes the set of known interactions between the miRNAs in *M* and the lncRNAs in *L*; $E_{3}=\left\{e_{l_{i}-d_{j}}|l_{i}\in L^{\prime },d_{j}\in D^{\prime }\right\}$ represents the set of interactions between the lncRNAs in $L^{\prime }$ and the diseases in $D^{\prime }$. Thus, the edge $e_{m_{k}-l_{i}}\in E_{2}\Leftrightarrow m_{k}$ is associated with $l_{i}$, and the edge $e_{l_{i}-d_{j}}\in E_{3}\Leftrightarrow l_{i}$ is associated with $d_{j}$. Finally, the global tripartite network, $GN_{1}$, is expressed as $GN_{1}=\left(L,D,M,E\right)$, where $E=E_{1}\cup E_{2}\cup E_{3}$.

### 3.2. Construction of GDN, GLN, GMN, and $GN_{2}$

Let $D^{\prime \prime }$ be the set of $r^{\prime \prime }$ diseases in ${\mathrm{DS}}_{4}$, $L^{\prime \prime }$ be the set of $n^{\prime \prime }$ lncRNAs in ${\mathrm{DS}}_{5}$, *G* be the set of *p* genes in ${\mathrm{DS}}_{4}$ or ${\mathrm{DS}}_{5}$, $G^{\prime }$ be the set of $p^{\prime }$ genes in ${\mathrm{DS}}_{6}$, and $M^{\prime }$ be the set of $t^{\prime }$ miRNAs in ${\mathrm{DS}}_{6}$. Additionally, from Section 2.3 and Section 2.4, it is clear that $D^{\prime \prime }\subseteq D$, $L^{\prime \prime }\subseteq L$, and $G^{\prime }\subseteq G$; hence, we can let $D^{\prime }=\left\{d_{1},d_{2},\ldots ,d_{r^{\prime \prime }}\right\}$, $L^{\prime }=\left\{l_{1},l_{2},\ldots ,l_{n^{\prime \prime }}\right\}$, $G^{\prime }=\left\{g_{1},g_{2},\ldots ,g_{p^{\prime }}\right\}$, $G=\{g_{1},g_{2},\ldots ,g_{p^{\prime }},g_{p^{\prime }+1},\ldots ,g_{p}\}$, and $M^{\prime }=\left\{m_{1},m_{2},\ldots ,m_{t^{\prime }}\right\}$. We can thus represent the gene–disease association network, GDN, as $GDN=(G,D,E_{4})$, where $E_{4}=\left\{e_{g_{f}-d_{j}}|g_{f}\in G,d_{j}\in D^{\prime \prime }\right\}$ denotes the set of known interactions between the genes in *G* and the diseases in $D^{\prime \prime }$. That is, the edge $e_{g_{f}-d_{j}}\in E_{4}\Leftrightarrow g_{f}$ is associated with $d_{j}$.

In the same way, we can further represent the gene–lncRNA interaction network, GLN, and gene–miRNA interaction network, GMN, as $GLN=(G,L,E_{5})$ and $GMN=(G,M,E_{6})$, where $E_{5}=\left\{e_{g_{f}-l_{i}}|g_{f}\in G,l_{i}\in L^{\prime \prime }\right\}$ and $E_{6}=\left\{e_{g_{f}-m_{k}}|g_{f}\in G^{\prime },m_{k}\in M^{\prime }\right\}$ denote the set of known gene–lncRNA interactions and the set of known gene–miRNA interactions, respectively. In other words, the edge $e_{g_{f}-l_{i}}\in E_{5}\Leftrightarrow g_{f}$ is associated with $l_{i}$ and the edge $e_{g_{f}-m_{k}}\in E_{6}\Leftrightarrow g_{f}$ is associated with $m_{k}$. Finally, it is evident that the global tripartite network $GN_{2}$ can be expressed as $GN_{2}=\left(L,D,M,G,E^{\prime }\right)$, where $E^{\prime }=E\cup E_{4}\cup E_{5}\cup E_{6}$.

### 3.3. Construction of NBCLDA

The naïve Bayesian classifier is a simple probabilistic classifier with a naïve independence assumption that any feature of a class is independent of the other features of the class. Abstractly, based on the Bayesian classifier probability model $p(C|F_{1},F_{2},\ldots ,F_{n})$, where *C* is a dependent class variable and $F_{1},F_{2},\ldots ,F_{n}$ are the feature variables of class *C*, the posterior probability can be described as follows:

(1) $$p\left(C|F_{1},F_{2},\ldots ,F_{n}\right)=\frac{p\left(F_{1},F_{2},\ldots ,F_{n}|C\right)p\left(C\right)}{p(F_{1},F_{2},\ldots ,F_{n})}.$$

Furthermore, according to the above assumption, since each feature $F_{i}$ is conditionally independent of every other feature $F_{j}(i\neq j)$, Equation (1) can be expressed as:

(2) $$p\left(C|F_{1},F_{2},\ldots ,F_{n}\right)=\frac{p\left(C\right)\prod_{i=1}^{n}p\left(F_{i}|C\right)}{p(F_{1},F_{2},\ldots ,F_{n})}.$$

**Figure 3:**
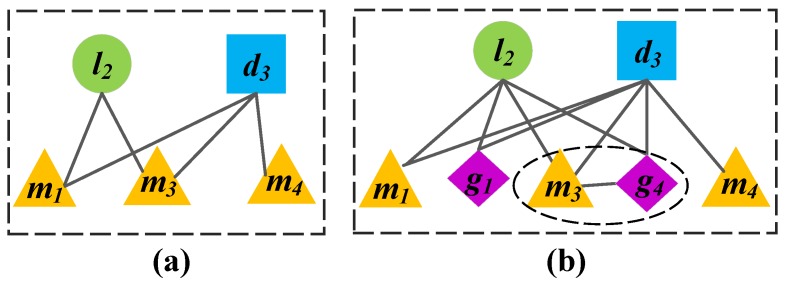
(**a**) a subnetwork of Figure 2b, in which, the common neighboring nodes $m_{1}$ and $m_{3}$ between $l_{2}$ and $d_{3}$, are assumed to be conditionally independent; (**b**) a subnetwork of Figure 2d, in which, $m_{1}$, $m_{3}$, $g_{1}$, and $g_{4}$ are the common neighboring nodes between $l_{2}$ and $d_{3}$. Here, $m_{3}$-$g_{4}$, $m_{1}$, and $g_{1}$ are assumed to be conditionally independent.

Inspired by existing probabilistic models based on Bayesian theory to predict missing links in complex networks [46], we designed a prediction model NBCLDA to infer potential disease-related lncRNAs; we applied the naïve Bayesian theory to $GN_{1}$ and $GN_{2}$, constructed in Section 3.1 and Section 3.2, respectively. In the context of Equation (1), in NBCLDA, the associations between lncRNAs and diseases in $GN_{1}$ and $GN_{2}$ are considered as the class of variables, while the common neighboring nodes of every lncRNA–disease pair in $GN_{1}$ and $GN_{2}$ are considered as the feature variables. In particular, when applying the naïve Bayesian theory to $GN_{1}$, for any given pair of lncRNA and disease nodes in $GN_{1}$, we will consider that their common neighboring miRNA nodes are all conditionally independent of each other, since all of the miRNAs are different, and, therefore, we assume that each of the miRNAs will not affect the others. To illustrate this assumption more intuitively, we provide an example in Figure 3a, in which the common neighboring nodes $m_{1}$ and $m_{3}$ between $l_{2}$ and $d_{3}$ will be assumed to be conditionally independent.

However, when applying the naïve Bayesian theory to $GN_{2}$, as there are two types of common neighboring nodes, miRNAs and genes, between a pair of lncRNA and disease nodes. In this case, it is unreasonable to consider that all of these common neighbors are conditionally independent of each other, since there may exist interactions between genes and miRNAs. Therefore, for any given pair of lncRNA and disease nodes in $GN_{2}$, let ϕ be the set that consists of all their common neighboring nodes. Then, for any miRNA node $m^{*}$, if there is a gene node $g^{*}$ that is associated with $m^{*}$, we will consider the miRNA $m^{*}$ and its related gene $g^{*}$ as a whole, and denote them as $m^{*}$-$g^{*}$ and label this an miRNA–gene pair. By this means, it is obvious that there will be three kinds of features in ϕ—miRNAs, genes, and miRNA–gene pairs. Hence, we assume that these three kinds of elements in ϕ are conditionally independent of each other. To illustrate this assumption more intuitively, we present an example in Figure 3b, in which, $m_{1}$, $m_{3}$, $g_{1}$, and $g_{4}$ are the common neighboring nodes between $l_{2}$ and $d_{3}$, and we will assume that $m_{3}$-$g_{4}$, $m_{1}$, and $g_{1}$ are conditionally independent of each other.

#### 3.3.1. Method for Applying the Naïve Bayesian Theory into $GN_{1}$

For any given lncRNA node $l_{i}$ and disease node $d_{j}$ in $GN_{1}$, let $N(l_{i})$ and $N(d_{j})$ be the sets of neighboring nodes that are directly connected to $l_{i}$ and $d_{j}$, respectively. From this, we construct $CN\left(l_{i},d_{j}\right)=\left\{m_{1},m_{2},\ldots ,m_{h}\right\}$, which denotes the set consisting of all common neighboring nodes between $l_{i}$ and $d_{j}$ in $GN_{1}$. Then, the prior probabilities for the existence of an relationship edge $e_{l_{i}-d_{j}}$ are calculated via:

(3) $$p\left(e_{l_{i}-d_{j}}=1\right)=\frac{|M^{c}|}{\left|M\right|},$$

(4) $$p\left(e_{l_{i}-d_{j}}=0\right)=1-p\left(e_{l_{i}-d_{j}}=1\right),$$

where $|M^{c}|$ denotes the number of known associations between lncRNAs and diseases in LDN, and |M|=n×r, where *n* denotes the number of lncRNAs in *L* and *r* denotes the number of diseases in *D*.

Based on the naïve Bayesian classifier, the posterior probabilities for an edge $e_{l_{i}-d_{j}}$, representing whether the node $l_{i}$ is connected to $d_{j}$ in $GN_{1}$, are defined as follows:

(5) $$\begin{array}{c} p\left(e_{l_{i}-d_{j}}=1|CN\left(l_{i},d_{j}\right)\right)=\frac{p(e_{l_{i}-d_{j}}=1)}{p(CN\left(l_{i},d_{j}\right))}\prod_{m_{\delta }\in CN\left(l_{i},d_{j}\right)}p\left(m_{\delta }|e_{l_{i}-d_{j}}=1\right), \end{array}$$

(6) $$p\left(e_{l_{i}-d_{j}}=0|CN\left(l_{i},d_{j}\right)\right)=\frac{p(e_{l_{i}-d_{j}}=0)}{p(CN\left(l_{i},d_{j}\right))}\prod_{m_{\delta }\in CN\left(l_{i},d_{j}\right)}p\left(m_{\delta }|e_{l_{i}-d_{j}}=0\right).$$

From Equations (5) and (6), we can directly identify whether an lncRNA node is connected with a disease node or not in $GN_{1}$. However, since it is often too complicated to calculate the value of $p(CN\left(l_{i},d_{j}\right))$, we first define the probability of a potential association existing between $l_{i}$ and $d_{j}$ in $GN_{1}$ as follows:

(7) $$S1\left(l_{i},d_{j}\right)=\frac{p(e_{l_{i}-d_{j}}=1)}{p(e_{l_{i}-d_{j}}=0)}\prod_{m_{\delta }\in CN\left(l_{i},d_{j}\right)}\frac{p(m_{\delta }|e_{l_{i}-d_{j}}=1)}{p(m_{\delta }|e_{l_{i}-d_{j}}=0)},$$

where $p(m_{\delta }|e_{l_{i}-d_{j}}=1)$ and $p(m_{\delta }|e_{l_{i}-d_{j}}=0)$ are the conditional probabilities of a node $m_{\delta }$ belonging to $CN(l_{i},d_{j})$; they represent the possibilities of whether the node is a common neighboring node between $l_{i}$ and $d_{j}$ in $GN_{1}$ or not, respectively. Moreover, according to Bayesian theory, these two conditional probabilities can be expressed as:

(8) $$p\left(m_{\delta }|e_{l_{i}-d_{j}}=1\right)=\frac{p\left(e_{l_{i}-d_{j}}=1|m_{\delta }\right)p\left(m_{\delta }\right)}{p(e_{l_{i}-d_{j}}=1)},$$

(9) $$p\left(m_{\delta }|e_{l_{i}-d_{j}}=0\right)=\frac{p\left(e_{l_{i}-d_{j}}=0|m_{\delta }\right)p\left(m_{\delta }\right)}{p(e_{l_{i}-d_{j}}=0)},$$

where $p(e_{l_{i}-d_{j}}=1|m_{\delta })$ and $p(e_{l_{i}-d_{j}}=0|m_{\delta })$ represent the conditional probability of whether the lncRNA node $l_{i}$ is connected to the disease node $d_{j}$ or not, respectively, and $m_{\delta }$ is one of the common neighboring nodes between $l_{i}$ and $d_{j}$ in $GN_{1}$. Thus, $p(e_{l_{i}-d_{j}}=1|m_{\delta })$ and $p(e_{l_{i}-d_{j}}=0|m_{\delta })$ are calculated via the following formulas:

(10) $$p\left(e_{l_{i}-d_{j}}=1|m_{\delta }\right)=\frac{N_{m_{\delta }}^{+}}{N_{m_{\delta }}^{+}+N_{m_{\delta }}^{-}},$$

(11) $$p\left(e_{l_{i}-d_{j}}=0|m_{\delta }\right)=\frac{N_{m_{\delta }}^{-}}{N_{m_{\delta }}^{+}+N_{m_{\delta }}^{-}},$$

where $N_{m_{\delta }}^{+}$ and $N_{m_{\delta }}^{-}$ denote the number of known and unknown associations between lncRNAs and diseases whose common neighbors include $m_{\delta }$, respectively.

Hence, from Equations (8) and (9), Equation (7) can be modified as follows:

(12) $$S1\left(l_{i},d_{j}\right)=\frac{p(e_{l_{i}-d_{j}}=1)}{p(e_{l_{i}-d_{j}}=0)}\prod_{m_{\delta }\in CN\left(l_{i},d_{j}\right)}\frac{p\left(e_{l_{i}-d_{j}}=0\right)p\left(e_{l_{i}-d_{j}}=1|m_{\delta }\right)}{p\left(e_{l_{i}-d_{j}}=1\right)p\left(e_{l_{i}-d_{j}}=0|m_{\delta }\right)}.$$

Moreover, given any two nodes $l_{i}$ and $d_{j}$ in $GN_{1}$, the value of $\frac{p(e_{l_{i}-d_{j}}=1)}{p(e_{l_{i}-d_{j}}=0)}$ is a constant, which we denote as $\phi_{m}$ for convenience. Additionally, for each common neighboring node between $l_{i}$ and $d_{j}$ in $GN_{1}$, let $N_{l}$ denote the number of lncRNAs directly related to $m_{\delta }$, and $N_{d}$ denote the number of diseases directly related to $m_{\delta }$. Then, $N_{m_{\delta }}^{+}+N_{m_{\delta }}^{-}=N_{l}\times N_{d}$, and hence, Equation (7) can further be modified as follows:

(13) $$S1\left(l_{i},d_{j}\right)=\phi_{m}\prod_{m_{\delta }\in CN\left(l_{i},d_{j}\right)}{\phi_{m}}^{-1}\frac{N_{m_{\delta }}^{+}}{N_{m_{\delta }}^{-}}.$$

Considering that $N_{m_{\delta }}^{+}$ may equal zero, we will introduce the Laplace calibration to guarantee that the value of $S1(l_{i},d_{j})$ will not be zero:

(14) $$S1\left(l_{i},d_{j}\right)=\phi_{m}\prod_{m_{\delta }\in CN\left(l_{i},d_{j}\right)}{\phi_{m}}^{-1}\frac{N_{m_{\delta }}^{+}+1}{N_{m_{\delta }}^{-}+1}.$$

Furthermore, by introducing the logarithmic function for standardization, for any given lncRNA node $l_{i}$ and disease node $d_{j}$ in $GN_{1}$, we can finally define the probability of a potential association existing between them as:

(15) $$S1^{\prime }\left(l_{i},d_{j}\right)=\frac{log(S1\left(l_{i},d_{j}\right))}{\lambda },$$

where λ is a constant utilized for normalization.

#### 3.3.2. Method for Applying the Naïve Bayesian Theory to $GN_{2}$

In the same manner as described in Section 3.3.1, for any given lncRNA node $l_{i}$ and disease node $d_{j}$ in $GN_{2}$, we construct the set consisting of all common neighboring nodes, $CN^{\prime }\left(l_{i},d_{j}\right)=\left\{m_{1},m_{2},\ldots ,m_{h},g_{1},g_{2},\ldots ,g_{u}\right\}$. Then, the posterior probabilities of $p^{\prime }(e_{l_{i}-d_{j}}=1|CN^{\prime }\left(l_{i},d_{j})\right)$ and $p^{\prime }(e_{l_{i}-d_{j}}=0|CN^{\prime }\left(l_{i},d_{j})\right)$, representing whether the node $l_{i}$ is connected to $d_{j}$ in $GN_{2}$ or not, respectively. Then, similarly as described in Section 3.3.1, we can define the probability of a potential association existing between $l_{i}$ and $d_{j}$ in $GN_{2}$ as follows (the deep representation of scheme are described in Supplementary Material):

(16) $$\begin{array}{c} S2\left(l_{i},d_{j}\right)=\phi_{m}\prod_{m_{\alpha }\in CN^{\prime }\left(l_{i},d_{j}\right)}\prod_{g_{\beta }\in CN^{\prime }\left(l_{i},d_{j}\right)}\prod_{m_{\bar{\alpha }},g_{\bar{\beta }}\in CN^{\prime }\left(l_{i},d_{j}\right)}{\phi_{m}}^{-3}\frac{\left(N_{m_{\alpha }}^{+}+1\right)\left(N_{g_{\beta }}^{+}+1\right)\left(N_{m_{\bar{\alpha }},g_{\bar{\beta }}}^{+}+1\right)}{\left(N_{m_{\alpha }}^{-}+1\right)\left(N_{g_{\beta }}^{-}+1\right)\left(N_{m_{\bar{\alpha }},g_{\bar{\beta }}}^{-}+1\right)}, \end{array}$$

where $N_{m_{\bar{\alpha }},g_{\bar{\beta }}}^{+}$ and $N_{m_{\bar{\alpha }},g_{\bar{\beta }}}^{-}$ denote the number of known and unknown associations between $l_{i}$ and $d_{j}$ in $GN_{2}$, respectively, conditional on $m_{\bar{\alpha }}$ and $g_{\bar{\beta }}$ being common neighboring nodes between $l_{i}$ and $d_{j}$ in $GN_{2}$ and $m_{\bar{\alpha }}$-$g_{\bar{\beta }}$ is an miRNA–gene pair. In addition, $N_{m_{\alpha }}^{+}$ and $N_{m_{\alpha }}^{-}$ denote the number of known and unknown associations between $l_{i}$ and $d_{j}$ in $GN_{2}$, respectively, conditional on $m_{\alpha }$ being a common neighboring node between $l_{i}$ and $d_{j}$. In addition, $N_{g_{\beta }}^{+}$ and $N_{g_{\beta }}^{-}$ represent the number of known and unknown associations between $l_{i}$ and $d_{j}$ in $GN_{2}$, respectively, conditional on $g_{\beta }$ being a common neighboring node between $l_{i}$ and $d_{j}$. Finally, following the example of Equation (15), we can finally define the probability of a potential association existing between $l_{i}$ and $d_{j}$ in $GN_{2}$ as follows:

(17) $$S2^{\prime }\left(l_{i},d_{j}\right)=\frac{log(S2\left(l_{i},d_{j}\right))}{\lambda }.$$

#### 3.3.3. Method of Appending the Disease Semantic Similarity into NBCLDA

The disease semantic similarity has been widely utilized as a valuable data source for discovering potential disease-related lncRNAs in many previous studies [30,38]. In this paper, we append the disease semantic similarity into our newly constructed prediction model NBCLDA to further uncover the potential relationships between lncRNAs and diseases.

From the description given in Section 2.5, we know that each disease term in the MeSH database can be described as a directed acyclic graph (DAG), in which the nodes represent the disease MeSH descriptors and all MeSH descriptors in the DAG are linked from more general terms (parent nodes) to more specific terms (child nodes) by a direct edge. Hence, in this paper, we first obtain the disease tree numbers according to the disease terms collected from the MeSH database. Thereafter, adopting the method proposed by Wang et al. [47], while supposing that disease $d_{j}$ is represented as the graph ${DAG}_{d_{j}}=\left(d_{j},T_{d_{j}},E_{d_{j}}\right)$, where $T_{d_{j}}$ is the set of all ancestor nodes of $d_{j}$ including node $d_{j}$, $E_{d_{j}}$ is the set of corresponding links, and the contribution of a disease *t* in ${DAG}_{d_{j}}$ to the semantic of disease $d_{j}$ can be calculated as follows:

(18) $$D_{d_{j}}\left(t\right)=\left\{\begin{array}{cc} 1, & \mathrm{if}\;t=d_{j},\\ max\{\Delta \times D_{d_{j}}\left(ct\right)|ct\in \mathrm{children}\;\mathrm{of}\;t\}, & \mathrm{if}\;t\neq d_{j}, \end{array}\right.$$

where Δ is the semantic contribution factor for edges $E_{d_{j}}$ linking disease $d_{j}$ with child disease t and the disease $d_{j}$ is the most specific disease and its own semantic score is defined as 1. Since nodes located farther from $d_{j}$ will be more general diseases that contribute less to $d_{j}$, then, based on Equation (24), we can define the semantic value of the disease $d_{j}$ as follows:

(19) $$DV\left(d_{j}\right)=\sum_{t\in T_{d_{j}}}D_{d_{j}}\left(t\right).$$

Therefore, based on the assumption that the diseases share the nodes of their DAGs, the semantic similarity between disease $d_{j}$ and $d_{i}$ can be defined as:

(20) $$SD\left(d_{j},d_{i}\right)=\frac{\sum_{t\in T_{d_{j}}\cap T_{d_{i}}}\left(D_{d_{j}}\left(t\right)+D_{d_{i}}\left(t\right)\right)}{DV\left(d_{j}\right)+DV\left(d_{i}\right)}.$$

Finally, based on the disease semantic similarity and the similarities between lncRNAs and diseases, we can reconstruct a new recommended measurement for inferring potential associations between lncRNAs and diseases as follows:

(21) $$S=S^{\prime }\times SD,$$

where $S^{\prime }$ denotes either $S1^{\prime }\left(l_{i},d_{j}\right)$ or $S2^{\prime }\left(l_{i},d_{j}\right)$ and SD, which is computed via Equation (20) denotes the disease semantic similarity.

## 4. Results

### 4.1. Performance Evaluation

The performance of the NBCLDA, for inferring potential associations between lncRNAs and diseases, is evaluated by implementing LOOCV and is based on experimentally verified lncRNA–disease associations. At each round, a known lncRNA–disease association is used as a test sample, whereas all the remaining associations are taken as training cases for model learning. This step continues until each sample is treated as a verification sample. Moreover, the value of area under the receiver operating characteristic (ROC) curve (AUC) can be applied for measuring the overall performance of the method. The closer the AUC value is to 1, the better the performance is, and an AUC value of 0.5 refers to a random guess. We calculate a series of true positive rates (TPR or sensitivity) and false positive rates (FPR or 1−specificity) by setting different classification thresholds, and the ROC curve is plotted with the functional relationship between them. Specifically, TPR corresponds to the ratio of the successfully predicted lncRNA–disease associations to the total experimentally verified lncRNA–disease associations, and FPR refers to the percentage of candidate lncRNAs ranked below the threshold.

**Figure 4:**
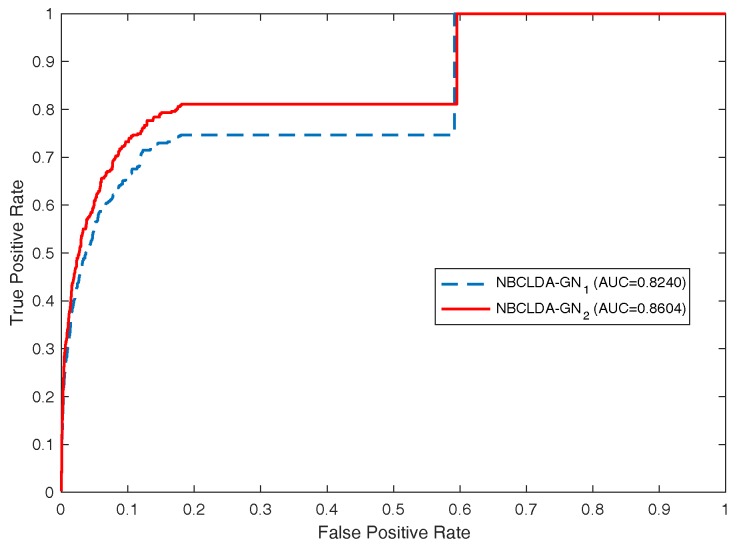
Performance evaluation for the NBCLDA in terms of ROC curves and AUCs based on the experimentally known associations (data set ${\mathrm{DS}}_{3}$), in the framework of LOOCV. Here, NBCLDA-$GN_{1}$ and NBCLDA-$GN_{2}$ represent the simulation results while implementing our algorithm on the global networks $GN_{1}$ and $GN_{2}$, respectively.

**Figure 5:**
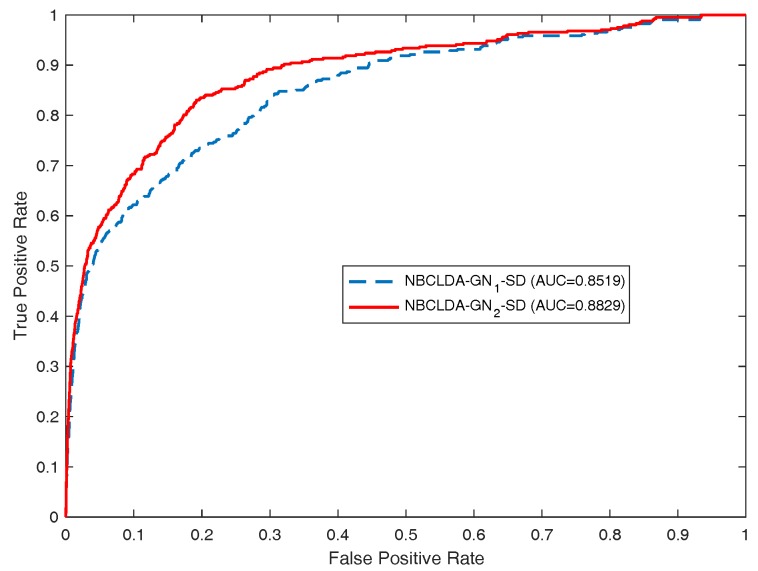
Same as Figure 4, but additionally including disease semantic similarity. Here, NBCLDA-$GN_{1}$-SD and NBCLDA-$GN_{2}$-SD represent the simulation results when appending the disease semantic similarity to the NBCLDA on networks $GN_{1}$ and $GN_{2}$, respectively.

First, in order to estimate the influence of the addition of new types of nodes and the introduction of the disease semantic similarity on the predictions of potential associations between lncRNAs and diseases, we implemented the NBCLDA on the two constructed global networks $GN_{1}$ and $GN_{2}$ in the framework of LOOCV. The simulation results are shown in Figure 4 and Figure 5. From Figure 4, the NBCLDA achieved an AUC of 0.8240 on $GN_{1}$ and an AUC of 0.8604 on $GN_{2}$ when the disease semantic similarity was not utilized. On the other hand, from Figure 5, an AUC of 0.8519 on $GN_{1}$ and an AUC of 0.8819 on $GN_{2}$ were achieved when the disease semantic similarity was included. This demonstrates that the prediction performance of our method not only benefits from the addition of the new types of nodes for predicting potential associations between lncRNAs and diseases, but also is significantly improved by the introduction of disease semantic similarity.

**Figure 6:**
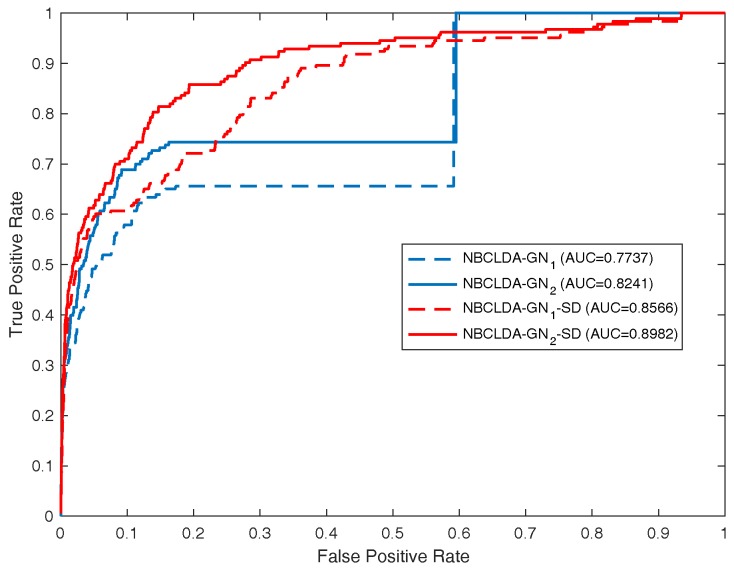
The performance of the NBCLDA in terms of ROC curves and AUCs based on 183 known lncRNA–disease associations, in the framework of the LOOCV.

In order to further assess the performance of the NBCLDA, we compared it with other state-of-the-art models including HGLDA [27], SIMCLDA [35], MFLDA [37], Yang et al. method [26], KATZLDA [38] and TPGLDA [26] in the framework of LOOCV. For comparing with the HGLDA, a data set consisting of 183 experimentally validated lncRNA–disease associations was previously constructed and taken as the test set to evaluate its performance. Hence, for convenience, we compared our model, the NBCLDA, with the HGLDA on that data set using the framework of LOOCV. The simulation results are illustrated in Table 1 and Figure 6, from which it is evident that our approach outperformed the HGLDA. For comparing with SIMCLDA, a data set consisting of 101 known lncRNA–disease associations between 30 lncRNAs and 79 diseases was collected from the data set containing of 293 experimentally validated lncRNA–disease associations which was used in method SIMCLDA. These selected lncRNAs and diseases all belong to ${\mathrm{DS}}_{3}$ in our paper. The simulation results are illustrated in Table 1, from which it is evident that our approach outperformed the SIMCLDA. While comparing with MFLDA, six relational data sources including lncRNA–miRNA associations, lncRNA–gene function associations, lncRNA–disease associations, miRNA–gene interactions, miRNA–disease associations and gene–disease associations, which were used in the method MFLDA, were collected to implement NBCLDA. The data set of experimentally validated lncRNA–disease associations was taken as the test set to evaluate its performance. The simulation results are illustrated in Table 1, from which it is evident that our approach outperformed the MFLDA.

**Figure 7:**
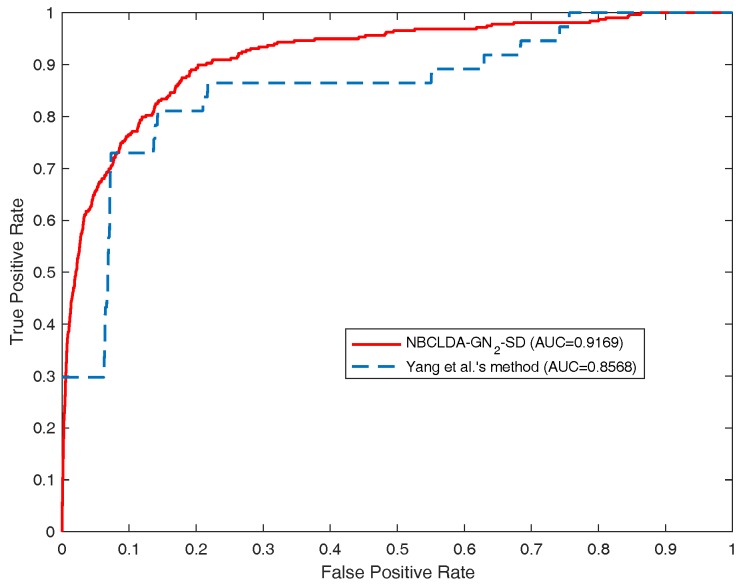
Comparison of the performance of the NBCLDA and Yang et al.’s method in terms of ROC curves and AUCs based on a data set of 319 lncRNA–disease associations between 37 lncRNAs and 52 diseases in the framework of the LOOCV.

**Figure 8:**
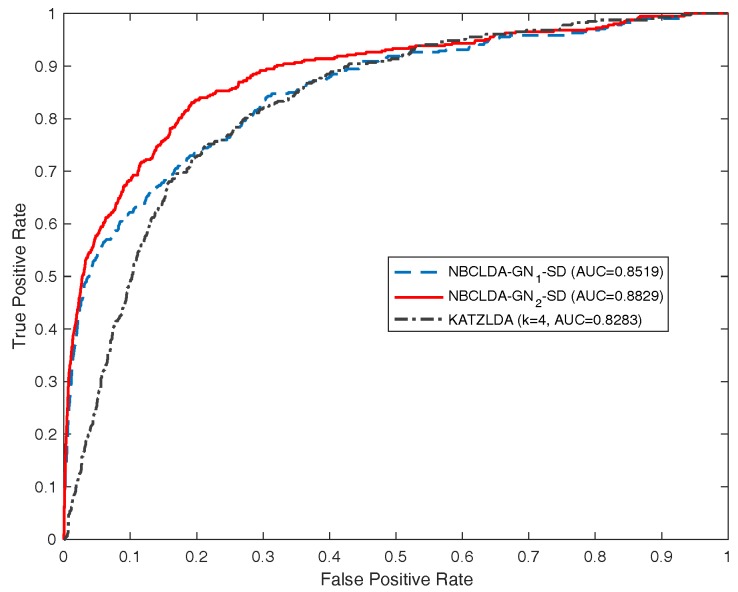
Comparison of the performance of the NBCLDA and KATZLDA approaches in terms of ROC curves and AUCs based on data set ${\mathrm{DS}}_{3}$, in the framework of the LOOCV.

Furthermore, we compared the NBCLDA with Yang et al.’s method based on the data set ${\mathrm{DS}}_{3}$ consisting of 407 lncRNA–disease associations between 77 lncRNAs and 95 diseases. In order to make a comparison with Yang et al.’s method, according to their description, we first deleted the nodes with a degree equal to 1. As a result, we obtained a data set consisting of 319 lncRNA–disease associations between 37 lncRNAs and 52 diseases. Then, we took this data set as the test set to compare the two methods in the framework of the LOOCV. The simulation results are shown in Figure 7, from which it is seen that the NBCLDA achieved an AUC of 0.9169 while being implemented on $GN_{2}$, which is much better than the AUC of 0.8568 achieved by Yang et al.’s method. We also compared the NBCLDA with the KATZLDA, which is a path-based method designed to predict potential lncRNA–disease associations by integrating multiple pieces of information including known lncRNA–disease associations, lncRNA expression profiles, lncRNA functional similarity, disease semantic similarity, and the Gaussian interaction profile kernel similarity. Executing the simulation, we could not obtain information on the expression profiles of corresponding lncRNAs; thus, we compared the two methods without this information. The simulation results are shown in Figure 8, which indicate that the NBCLDA achieves higher AUCs (of 0.8519 and 0.8829) than the KATZLDA with a corresponding AUC of 0.8323. This also demonstrates the superiority of our newly constructed prediction model, the NBCLDA. Finally, comparing with TPGLDA, a data set consisting of 312 experimentally validated lncRNA–disease associations including 68 lncRNAs and 67 diseases and a data set consisting of 1941 gene–disease associations between 165 genes and 67 diseases were constructed, respectively. The data set of known lncRNA–disease associations was taken as the test set to evaluate its performance. The simulation results are illustrated in Table 1, from which it is obvious that TPGLDA can achieve a better performance with an AUC of 0.92, which is higher than that of ours with the AUC value of 0.8982. The main reason that TPGLDA can achieve a better performance is probably that the contribution of resource moved in both directions are taken into consideration by a consistence-based resource allocation algorithm. However, NBCLDA does not entirely rely on known lncRNA–disease associations and can integrate multiple data sources to predict potential associations.

In order to further evaluate the performance of NBCLDA, 20 percent of the known lncRNA–disease associations are randomly chosen as training set, while the remaining known and all the unknown associations are taken as testing set. We then compare with the six methods on the predicted top-k associations by using F1-score measure, which is a measure of a test’s accuracy [48]. Since the sparse known lncRNA–disease associations, we set different threshold *k* based on the different set of known associations when comparing with other methods and the comparison results are illustrated in Table 2. From Table 2, we could see that NBCLDA outperforms several other methods in terms of F1-score. However, TPGLDA could achieve higher values than that of our approach, this is likely due to that resource moved in both directions are taken into consideration by consistence-based resource allocation algorithm. However, comparing with TPGLDA, our new method does not entirely rely on known lncRNA–disease associations and can integrate multiple data sources to predict potential associations. These advantages may be an excellent addition for biomedical research in the future.

### 4.2. Case Studies

To further estimate the performance of the NBCLDA, case studies of three types of lncRNA-related diseases—colorectal cancer, prostate cancer, and glioma—are analyzed in this section. During the simulation experiment, the known lncRNA–disease associations in the data set ${\mathrm{DS}}_{3}$ were considered as the training samples, while the experimentally validated lncRNA–disease associations beyond ${\mathrm{DS}}_{3}$ were used for testing. As for the simulation results, the top 20 disease-related lncRNAs, predicted by the NBCLDA, were verified via relevant literature, and the corresponding evidence is listed in Table 3. In addition, the predicted results of the top 20 disease-related lncRNAs were presented in the Supplementary Table S8.

Colorectal cancer (CRC) is one of the most common cancer types in western countries and its morbidity increases with age [49]. Accumulating studies have shown that lncRNAs play important roles in several steps of carcinogenesis and cancer metastasis and additionally interact with various cancers including CRC [50,51]. Therefore, we implemented the NBCLDA to discover possible CRC-associated lncRNAs. As illustrated in Table 3, seven of the top 20 lncRNAs have been validated to be related to colorectal cancer by recent biological literature, and five of them are ranked in the top 10 of the prioritized prediction results. The other two are lncRNAs SNHG16 (ranked 12th) and TUG1 (ranked 18th). For example, Chen et al. indicated that the lncRNA XIST can regulate the process of CRC development by competing for miR-200b-3p and thus it may be considered as a biomarker for prognosis [52]. Additionally, it has been demonstrated that the lncRNA MALAT1 may be considered as a potential prognostic and therapeutic target of colorectal cancer patients as it can fulfill a chemoresistant function in colorectal cancer [53]. Nakano et al. found that the epigenetic destruction and loss of imprinting of the lncRNA KCNQ1OT1 play a significant role in the occurrence of colorectal cancer [54]. Han et al. suggested that H19 can be considered as a candidate therapeutic biomarker and a new target for human CRC therapy when it is used as a growth regulator [55].

Prostate cancer is the second most common cause of cancer-related mortality in males worldwide [56]. Increasing studies show that lncRNA have become a promising target for the treatment of cancers including prostate cancer [57,58]. Hence, we carried out the NBCLDA to uncover possible prostate cancer-associated lncRNAs, and five of the top 20 predicted lncRNAs were verified and are listed in Table 3 according to the relevant literature. For example, Ren et al. evaluated the expression of MALAT1 in prostate cancer and showed that it may be considered as a perspective therapeutic target for refractory prostate cancer [59]. Zhu et al. found that the lncRNA H19 and its derived miRNA H19-miR-675 were significantly downregulated in advanced prostate cancer and they may be used for diagnostic and therapeutic treatment in advanced prostate cancer because H19-miR-675 could act as a suppressor of prostate cancer metastasis [60]. Additionally, Tian et al. showed that targeting the lncRNA NEAT1 axis could be used as a potential application in improving chemotherapy of prostate cancer [61].

Glioma is one of the most common malignant forms of brain tumors, and 6 out of 100,000 people may have gliomas [62]. Accumulating research has shown that lncRNAs play a significant role in the process of glioma development [63]. Therefore, we applied the NBCLDA to predict potential lncRNAs associated with glioma. Four of the top 20 glioma-related lncRNAs were validated by recent literature on biological experiments, and the results are illustrated in Table 3. For example, the lncRNA MALAT1 plays an important role in the progression and therapy of glioma and it may be considered an effective prognostic biomarker for the treatment of glioma [64]. Zhang et al. demonstrated that the lncRNA H19 was overexpressed in glioma tissue and cell lines, and also promotes cell proliferation of glioma [65]. Furthermore, Li et al. suggested that the lncRNA TUG1 can promote cell apoptosis of glioma cells and may act as a tumor suppressor in human glioma [66].

## 5. Discussion

Accumulating studies have indicated that lncRNAs play crucial roles in biological processes, complex disease diagnoses, prognoses, and treatments. Furthermore, computational models for predicting novel lncRNA–disease associations by integrating varieties of biological data are among the most noticeable topics. This is helpful to explore the understanding of disease mechanisms at the lncRNA level. In this paper, we construct a global tripartite network and a quadruple network by integrating various biological information and propose a novel approach, the NBCLDA, to predict potential lncRNA–disease associations by applying the naïve Bayesian classifier into the two constructed networks. Compared with current models, the NBCLDA does not entirely rely on known lncRNA–disease associations, and can achieve a reliable performance with effective AUCs in the LOOCV framework. This means that our method can not only predict the possible associations between lncRNAs and diseases included in the known associations set, but can also predict the potential associations whose elements are not in the known data set.

To evaluate the predictive performance of our method, the LOOCV is implemented based on the experimentally verified lncRNA–disease associations obtained from the MNDR database. Simulation experiment results of the NBCLDA show a strong performance and its predictive accuracy has been significantly improved by the addition of new types of nodes and the disease semantic similarity for predicting potential associations between lncRNAs and diseases. It also shows that the NBCLDA can achieve better performance than the other three state-of-the-art models with more effective AUCs in the framework of the LOOCV. Moreover, in order to further estimate the performance of the NBCLDA, case studies of colorectal cancer, prostate cancer, and glioma were implemented in this paper. These simulation results demonstrated that the NBCLDAs can be an excellent tool for future biomedical research.

Despite the reliable experimental results of the NBCLDA, there are also some biases in our method. For example, the known experimentally validated lncRNA–disease associations are still limited. Therefore, the prediction performance of the NBCLDA would be improved by a more comprehensive data set. Furthermore, the data sources in this paper need to be strictly preprocessed according to the proposed method, which restricts the richness of the data sources to a certain extent.

## 6. Conclusions

In this paper, we mainly summed up the following contributions: (1) we constructed a global tripartite network by integrating a variety of biological information including miRNA-disease, miRNA-lncRNA and lncRNA-diseases associations and interactions; (2) we constructed a global quadruple network by appending gene–lncRNA interaction, gene–disease association, and gene–miRNA interaction networks to the global tripartite network; (3) we developed a novel approach NBCLDA based on the naïve Bayesian classifier and applied it into the two global networks to predict potential lncRNA–disease associations; (4) we appended the disease semantic similarity into our newly constructed prediction model NBCLDA to further uncover the potential relationships between lncRNAs and diseases; (5) NBCLDA can not only predict the possible associations between lncRNAs and diseases included in the known associations set, but can also predict the potential associations whose elements are not in the known data set; (6) NBCLDA can integrate multiple heterogeneous biological data for discovering potential relationships between lncRNAs and diseases; (7) in the future work, more biological data can be collected and pre-processed to be utilized in the newly proposed method for predicting potential lncRNA-disease associations.

## Figures and Tables

**Table 1:** Performance comparisons between the NBCLDA and other state-of-the-art models in terms of AUCs based on the different data sets of known lncRNA–disease associations in the framework of the LOOCV.

Methods	AUCs	Methods	AUCs
NBCLDA-$GN_{2}$-SD	0.8982	NBCLDA-$GN_{2}$-SD	0.9169
HGLDA	0.7621	Yang et al. method	0.8568
NBCLDA-$GN_{2}$-SD	0.8897	NBCLDA-$GN_{2}$-SD	0.8829
SIMCLDA	0.8526	KATZLDA	0.8283
NBCLDA-$GN_{2}$-SD	0.8704	NBCLDA-$GN_{2}$-SD	0.8897
MFLDA	0.7945	TPGLDA	0.92

**Table 2:** F1-scores of NBCLDA, SIMCLDA, MFLDA, Yang et al.’s method, KATZLDA, TPGLDA at different top-*k* cutoffs

Methods		F1-Score	
NBCLDA	0.1536 (k = 20)	0.1582 (k = 40)	null (k = 60)
SIMCLDA	0.0635 (k = 20)	0.0482 (k = 40)	null (k = 60)
NBCLDA	0.1773 (k = 20)	0.2415 (k = 40)	null (k = 60)
MFLDA	0.2012 (k = 20)	0.1139 (k = 40)	null (k = 60)
NBCLDA	0.2575 (k = 20)	0.2855 (k = 34)	null (k = 60)
Yang et al.’s method	0.2707 (k = 20)	0.2769 (k = 34)	null (k = 60)
NBCLDA	0.1183 (k = 20)	0.1088 (k = 40)	0.1139 (k = 60)
KATZLDA	0.1274 (k = 20)	0.0869 (k = 40)	0.0779 (k = 60)
NBCLDA	0.1295 (k = 20)	0.1510 (k = 40)	0.1320 (k = 60)
TPGLDA	0.2070 (k = 20)	0.1644 (k = 40)	0.1301 (k = 60)

**Table 3:** The lncRNAs in the top 20 for the three case studies.

Disease	lncRNA	Evidence (PMID)	Rank
Colorectal cancer	XIST	17143621	1
Colorectal cancer	MALAT1	25446987,25031737,21503572,25025966,24244343,26887056	3
Colorectal cancer	KCNQ1OT1	16965397	6
Colorectal cancer	H19	11120891,19926638,22427002,26068968,26989025	8
Colorectal cancer	NEAT1	26314847	9
Colorectal cancer	SNHG16	24519959	12
Colorectal cancer	TUG1	26856330	18
Prostate cancer	MALAT1	23845456,23726266,26516927,22349460	3
Prostate cancer	KCNQ1OT1	23728290	6
Prostate cancer	H19	24063685,24988946	8
Prostate cancer	NEAT1	23728290,25415230	10
Prostate cancer	TUG1	26975529	19
Glioma	MALAT1	26649278,25613066,26619802,27134488,26938295	4
Glioma	H19	24466011,26983719	6
Glioma	TUG1	25645334,27363339	10
Glioma	NEAT1	26582084	12

## Supplementary Materials

The following are available at http://www.mdpi.com/2073-4425/9/7/345/s1, Supplementary Table S1: The known miRNA–disease associations of the data set ${\mathrm{DS}}_{1}$ consisting of 4704 miRNA–disease interactions which were collected from the HMDD database; Supplementary Table S2: The known miRNA–lncRNA associations of the data set ${\mathrm{DS}}_{2}$ consisting of 9086 miRNA–lncRNA interactions which were collected from the starBase v2.0 database; Supplementary Table S3: The known lncRNA–disease associations of the data set ${\mathrm{DS}}_{3}$ consisting of 407 lncRNA–disease associations which were downloaded from the MNDR v2.0 database; Supplementary Table S4: The known gene–disease associations of the data set ${\mathrm{DS}}_{4}$ consisting of 3702 gene–disease associations which were gathered from the DisGeNET v5.0 database; Supplementary Table S5: The known gene–lncRNA associations of the data set ${\mathrm{DS}}_{5}$ consisting of 411 gene–lncRNA interactions which were downloaded from the LncACTdb database; Supplementary Table S6: The known gene–miRNA associations of the data set ${\mathrm{DS}}_{6}$ consisting of 565 gene–miRNA association was obtained from the miRecords database; Supplementary Table S7: The Disease tree numbers of the data set ${\mathrm{DS}}_{7}$ consisting of 373 diseases with their disease tree numbers which were gathered from the MeSH database; Supplementary Table S8: The results of top 20 lncRNAs related to these three diseases. Supplementary Materials: Deep representation of the probabilistic scheme in our method.
