# Supplementary material for: A Novel Probability Model for LncRNA–Disease Association Prediction Based on the Naïve Bayesian Classifier
Source: Genes (Basel). 2018 Jul 8;9(7):345. doi: 10.3390/genes9070345 (PMC6071012; doi:10.3390/genes9070345)
Supplement: Supplementary file 1 [file genes-09-00345-s001.zip › Supplementary Material.pdf]

Article

A novel probability model for LncRNA-Disease Association Prediction based on the Naïve Bayesian Classifier

Jingwen Yu [1], Pengyao Ping [1], Lei Wang [1,2*], Linai Kuang [1,2], Xueyong Li [2] and Zhelun Wu[3]

1 Key Laboratory of Intelligent Computing & Information Processing, Xiangtan University. XiangTan 411105, People's Republic of China; jingwen-yu@qq.com; pengyao.ping@qq.com; wanglei@xtu.edu.cn; kla@xtu.edu.cn
2 College of Computer Engineering & Applied Mathematics, Changsha University, Changsha 410001, Hunan, People's Republic of China; wanglei@xtu.edu.cn; kla@xtu.edu.cn; 137493255@qq.com
3 Department of Computer Science, Princeton University, Princeton, New Jersey, USA; zhelunw@princeton.edu
* Correspondence: wanglei@xtu.edu.cn; Tel.: +86-151-1110-9999

Academic Editor: name
Version June 24, 2018 submitted to Journal Not Specified

1. Method

1.1. Method for Applying the Naïve Bayesian Theory into GN_1

Based on the naïve Bayesian classifier, the posterior probabilities for an edge $e_{l_i-d_j}$, representing whether the node l_i is connected to d_j in GN_1, are defined as follows:

$$\begin{aligned} p(e_{l_i-d_j}=1|CN(l_i,d_j)) &= \frac{p(m_1,m_2,\ldots,m_h|e_{l_i-d_j}=1)p(e_{l_i-d_j}=1)}{p(CN(l_i,d_j))} \\ &= \frac{p(e_{l_i-d_j}=1)}{p(CN(l_i,d_j))}\prod_{m_\delta\in CN(l_i,d_j)} p(m_\delta|e_{l_i-d_j}=1) \end{aligned} \tag{1}$$

$$\begin{aligned} p(e_{l_i-d_j}=0|CN(l_i,d_j)) &= \frac{p(m_1,m_2,\ldots,m_h|e_{l_i-d_j}=0)p(e_{l_i-d_j}=1)}{p(CN(l_i,d_j))} \\ &= \frac{p(e_{l_i-d_j}=0)}{p(CN(l_i,d_j))}\prod_{m_\delta\in CN(l_i,d_j)} p(m_\delta|e_{l_i-d_j}=0). \end{aligned} \tag{2}$$

From Equations (1) and (2), we can directly identify whether an lncRNA node is connected with a disease node or not in GN_1. However, since it is often too complicated to calculate the value of $p(CN(l_i,d_j))$, we first define the probability of a potential association existing between l_i and d_j in GN_1 as follows:

$$S1(l_i,d_j) = \frac{p(e_{l_i-d_j}=1|CN(l_i,d_j))}{p(e_{l_i-d_j}=0|CN(l_i,d_j))} = \frac{p(e_{l_i-d_j}=1)}{p(e_{l_i-d_j}=0)}\prod_{m_\delta\in CN(l_i,d_j)}\frac{p(m_\delta|e_{l_i-d_j}=1)}{p(m_\delta|e_{l_i-d_j}=0)}, \tag{3}$$

where $p(m_\delta|e_{l_i-d_j}$=1) and $p(m_\delta|e_{l_i-d_j}$=0) are the conditional probabilities of a node m_δ belonging to $CN(l_i,d_j)$; they represent the possibilities of whether the node is a common neighboring node between

l_i and d_j in GN_1 or not, respectively. Moreover, according to Bayesian theory, these two conditional probabilities can be expressed as:

$$p(m_\delta | e_{l_i - d_j} = 1) = \frac{p(e_{l_i - d_j} = 1 | m_\delta) p(m_\delta)}{p(e_{l_i - d_j} = 1)} \tag{4}$$

$$p(m_\delta | e_{l_i - d_j} = 0) = \frac{p(e_{l_i - d_j} = 0 | m_\delta) p(m_\delta)}{p(e_{l_i - d_j} = 0)}, \tag{5}$$

where $p(e_{l_i - d_j}{=}1 | m_\delta)$ and $p(e_{l_i - d_j}{=}0 | m_\delta)$ represent the conditional probability of whether the lncRNA node l_i is connected to the disease node d_j or not, respectively, and m_δ is one of the common neighboring nodes between l_i and d_j in GN_1. Thus, $p(e_{l_i - d_j}{=}1 | m_\delta)$ and $p(e_{l_i - d_j}{=}0 | m_\delta)$ are calculated via the following formulas:

$$p(e_{l_i - d_j} = 1 | m_\delta) = \frac{N^+_{m_\delta}}{N^+_{m_\delta} + N^-_{m_\delta}} \tag{6}$$

$$p(e_{l_i - d_j} = 0 | m_\delta) = \frac{N^-_{m_\delta}}{N^+_{m_\delta} + N^-_{m_\delta}}, \tag{7}$$

where $N^+_{m_\delta}$ and $N^-_{m_\delta}$ denote the number of known and unknown associations between lncRNAs and diseases whose common neighbors include m_δ respectively.

Hence, from Equations (4) and (5), Equation (3) can be modified as follows:

$$S1(l_i, d_j) = \frac{p(e_{l_i - d_j} = 1)}{p(e_{l_i - d_j} = 0)} \prod_{m_\delta \in CN(l_i, d_j)} \frac{p(e_{l_i - d_j} = 0) p(e_{l_i - d_j} = 1 | m_\delta)}{p(e_{l_i - d_j} = 1) p(e_{l_i - d_j} = 0 | m_\delta)}. \tag{8}$$

Moreover, given any two nodes l_i and d_j in GN_1, the value of $\frac{p(e_{l_i - d_j}=1)}{p(e_{l_i - d_j}=0)}$ is a constant, which we denote as ϕ_m for convenience. Additionally, for each common neighboring node between l_i and d_j in GN_1, let N_l denote the number of lncRNAs directly related to m_δ, and N_d denote the number of diseases directly related to m_δ. Then, $N^+_{m_\delta} + N^-_{m_\delta} = N_l \times N_d$, and hence, Equation (3) can further be modified as follows:

$$S1(l_i, d_j) = \phi_m \prod_{m_\delta \in CN(l_i, d_j)} {\phi_m}^{-1} \frac{N^+_{m_\delta}}{N^-_{m_\delta}}. \tag{9}$$

Considering that $N^+_{m_\delta}$ may equal zero, we will introduce the Laplace calibration to guarantee that the value of $S1(l_i, d_j)$ will not be zero:

$$S1(l_i, d_j) = \phi_m \prod_{m_\delta \in CN(l_i, d_j)} {\phi_m}^{-1} \frac{N^+_{m_\delta} + 1}{N^-_{m_\delta} + 1}. \tag{10}$$

Furthermore, by introducing the logarithmic function for standardization, for any given lncRNA node l_i and disease node d_j in GN_1, we can finally define the probability of a potential association existing between them as:

$$S1^{'}(l_i, d_j) = \frac{log(S1(l_i, d_j))}{\lambda}, \tag{11}$$

where, λ is a constant utilized for normalization.

1.2. Method for Applying the Naïve Bayesian Theory to GN_2

In the same manner as described in section 1.1, for any given lncRNA node l_i and disease node d_j in GN_2, we construct the set consisting of all common neighboring nodes, $CN'(l_i, d_j) = \{m_1, m_2, ..., m_h, g_1, g_2, ..., g_u\}$. Then, the posterior probabilities of $p'(e_{l_i - d_j}{=}1 | CN'(l_i, d_j))$

and $p'(e_{l_i-d_j}{=}0|CN'(l_i,d_j))$, representing whether the node l_i is connected to d_j in GN_2 or not, respectively, can be described as follows:

$$\begin{aligned}
p'(e_{l_i-d_j}=1|CN'(l_i,d_j)) &= \frac{p'(m_1,m_2,\ldots,m_h,g_1,g_2,\ldots,g_u|e_{l_i-d_j}=1)p'(e_{l_i-d_j}=1)}{p'(CN'(l_i,d_j))} \\
&= \frac{p'(e_{l_i-d_j}=1)}{p'(CN'(l_i,d_j))} \prod_{m_\alpha \in CN'(l_i,d_j)} p'(m_\alpha|e_{l_i-d_j}=1)\times \\
&\prod_{g_\beta \in CN'(l_i,d_j)} p'(g_\beta|e_{l_i-d_j}=1) \times \prod_{m_{\bar{\alpha}},g_{\bar{\beta}} \in CN'(l_i,d_j)} p'(m_{\bar{\alpha}},g_{\bar{\beta}}|e_{l_i-d_j}=1)
\end{aligned} \tag{12}$$

$$\begin{aligned}
p'(e_{l_i-d_j}=0|CN'(l_i,d_j)) &= \frac{p'(m_1,m_2,\ldots,m_h,g_1,g_2,\ldots,g_u|e_{l_i-d_j}=0)p'(e_{l_i-d_j}=0)}{p'(CN'(l_i,d_j))} \\
&= \frac{p'(e_{l_i-d_j}=0)}{p'(CN'(l_i,d_j))} \prod_{m_\alpha \in CN'(l_i,d_j)} p'(m_\alpha|e_{l_i-d_j}=0)\times \\
&\prod_{g_\beta \in CN'(l_i,d_j)} p'(g_\beta|e_{l_i-d_j}=0) \times \prod_{m_{\bar{\alpha}},g_{\bar{\beta}} \in CN'(l_i,d_j)} p'(m_{\bar{\alpha}},g_{\bar{\beta}}|e_{l_i-d_j}=0),
\end{aligned} \tag{13}$$

where $p'(m_\alpha|e_{l_i-d_j}{=}1)$ and $p'(m_\alpha|e_{l_i-d_j}{=}0)$ are the conditional probabilities of whether a node m_α belongs to $CN^{'}(l_i,d_j)$; they represent the possibilities of whether the node m_α is a common neighboring node between l_i and d_j in GN_2 or not, respectively. $p'(g_\beta|e_{l_i-d_j}{=}1)$ and $p'(g_\beta|e_{l_i-d_j}{=}0)$ are the conditional probabilities of whether a node g_β belonging to $CN^{'}(l_i,d_j)$; they represent the possibilities of whether the node g_β is a common neighboring node between l_i and d_j in GN_2 or not, respectively.

Following the example of Equations (4) and (5), we have:

$$p'(m_\alpha|e_{l_i-d_j}=1) = \frac{p'(e_{l_i-d_j}=1|g_\alpha)p'(g_\alpha)}{p'(e_{l_i-d_j}=1)} \tag{14}$$

$$p'(g_\alpha|e_{l_i-d_j}=0) = \frac{p'(e_{l_i-d_j}=0|g_\alpha)p'(g_\alpha)}{p'(e_{l_i-d_j}=0)} \tag{15}$$

$$p'(g_\beta|e_{l_i-d_j}=1) = \frac{p'(e_{l_i-d_j}=1|g_\beta)p'(g_\beta)}{p'(e_{l_i-d_j}=1)} \tag{16}$$

$$p'(g_\beta|e_{l_i-d_j}=0) = \frac{p'(e_{l_i-d_j}=0|g_\beta)p'(g_\beta)}{p'(e_{l_i-d_j}=0)} \tag{17}$$

$$p'(m_{\bar{\alpha}},g_{\bar{\beta}}|e_{l_i-d_j}=1) = \frac{p'(e_{l_i-d_j}=1|m_{\bar{\alpha}},g_{\bar{\beta}})p'(m_{\bar{\alpha}},g_{\bar{\beta}})}{p'(e_{l_i-d_j}=1)} \tag{18}$$

$$p'(m_{\bar{\alpha}},g_{\bar{\beta}}|e_{l_i-d_j}=0) = \frac{p'(e_{l_i-d_j}=0|m_{\bar{\alpha}},g_{\bar{\beta}})p'(m_{\bar{\alpha}},g_{\bar{\beta}})}{p'(e_{l_i-d_j}=0)}, \tag{19}$$

where $p'(e_{l_i-d_j}{=}1|m_{\bar{\alpha}},g_{\bar{\beta}})$ and $p'(e_{l_i-d_j}{=}0|m_{\bar{\alpha}},g_{\bar{\beta}})$ represent the conditional probability of whether the lncRNA node l_i is connected to the disease node d_j or not respectively, while both $m_{\bar{\alpha}}$ and $g_{\bar{\beta}}$ are common neighboring nodes between l_i and d_j in GN_2. Moreover, let $N^{+}_{m_{\bar{\alpha}},g_{\bar{\beta}}}$ and $N^{-}_{m_{\bar{\alpha}},g_{\bar{\beta}}}$ denote the number of known and unknown associations between l_i and d_j in GN_2, respectively, conditional on $m_{\bar{\alpha}}$ and $g_{\bar{\beta}}$ being common neighboring nodes between l_i and d_j in GN_2 and $m_{\bar{\alpha}}$-$g_{\bar{\beta}}$ is an miRNA–gene

pair. In addition, for any given miRNA–gene pair $m_{\bar{\alpha}}$-$g_{\bar{\beta}}$, let $N^{'}_{l_1}$ denote the number of lncRNAs directly related to $m_{\bar{\alpha}}$-$g_{\bar{\beta}}$, and $N^{'}_{d_1}$ denote the number of diseases directly related to $m_{\bar{\alpha}} - g_{\bar{\beta}}$; then, $N^{+}_{m_{\bar{\alpha}},g_{\bar{\beta}}} + N^{-}_{m_{\bar{\alpha}},g_{\bar{\beta}}} = N^{'}_{l_1} \times N^{'}_{d_1}$ and $p'(e_{l_i-d_j}{=}1|m_{\bar{\alpha}}, g_{\bar{\beta}}) + p'(e_{l_i-d_j}{=}0|m_{\bar{\alpha}}, g_{\bar{\beta}}){=}1$. Therefore, we have:

$$p^{'}(e_{l_i-d_j} = 1|m_{\bar{\alpha}}, g_{\bar{\beta}}) = \frac{N^{+}_{m_{\bar{\alpha}},g_{\bar{\beta}}}}{N^{+}_{m_{\bar{\alpha}},g_{\bar{\beta}}} + N^{-}_{m_{\bar{\alpha}},g_{\bar{\beta}}}}. \tag{20}$$

$$p^{'}(e_{l_i-d_j} = 0|m_{\bar{\alpha}}, g_{\bar{\beta}}) = \frac{N^{-}_{m_{\bar{\alpha}},g_{\bar{\beta}}}}{N^{+}_{m_{\bar{\alpha}},g_{\bar{\beta}}} + N^{-}_{m_{\bar{\alpha}},g_{\bar{\beta}}}}. \tag{21}$$

As illustrated in Section 1.1, we can define the probability of a potential association existing between l_i and d_j in GN_2 as follows:

$$S2(l_i, d_j) = \phi_m \prod_{m_\alpha \in CN'(l_i,d_j)} \prod_{g_\beta \in CN'(l_i,d_j)} \prod_{m_{\bar{\alpha}},g_{\bar{\beta}} \in CN'(l_i,d_j)} \phi_m{}^{-3} \frac{(N^{+}_{m_\alpha}+1)(N^{+}_{g_\beta}+1)(N^{+}_{m_{\bar{\alpha}},g_{\bar{\beta}}}+1)}{(N^{-}_{m_\alpha}+1)(N^{-}_{g_\beta}+1)(N^{-}_{m_{\bar{\alpha}},g_{\bar{\beta}}}+1)}, \tag{22}$$

where $N^{+}_{m_\alpha}$ and $N^{-}_{m_\alpha}$ denote the number of known and unknown associations between l_i and d_j in GN_2 respectively, conditional on m_α being a common neighboring node between l_i and d_j. In addition, $N^{+}_{g_\beta}$ and $N^{-}_{g_\beta}$ represent the number of known and unknown associations between l_i and d_j in GN_2 respectively, conditional on g_β being a common neighboring node between l_i and d_j. From the above descriptions, we find $N^{+}_{m_\alpha} + N^{-}_{m_\alpha} = N^{'}_{l_2} \times N^{'}_{d_2}$ and $N^{+}_{g_\beta} + N^{-}_{g_\beta} = N^{'}_{l_3} \times N^{'}_{d_3}$, where $N^{'}_{l_2}$ denotes the number of lncRNAs directly related to the node m_α in GN_2, $N^{'}_{d_2}$ denotes the number of diseases directly related to the node m_α in GN_2, $N^{'}_{l_3}$ denotes the number of lncRNAs directly related to the node g_β in GN_2, and $N^{'}_{d_3}$ denotes the number of diseases directly related to the node in GN_2.

Finally, following the example of Equation (11), we can finally define the probability of a potential association existing between l_i and d_j in GN_2 as follows:

$$S2^{'}(l_i, d_j) = \frac{log(S2(l_i, d_j))}{\lambda}. \tag{23}$$

© 2018 by the authors. Submitted to *Journal Not Specified* for possible open access publication under the terms and conditions of the Creative Commons Attribution (CC BY) license (http://creativecommons.org/licenses/by/4.0/).
